# Bis(2-methyl­imidazolium) fumarate dihydrate

**DOI:** 10.1107/S160053680903952X

**Published:** 2009-10-03

**Authors:** Zhiyuan Xie

**Affiliations:** a School of Physics and Electronic Engineering, Xiangfan University, Xiangfan 441053, Hubei, People’s Republic of China

## Abstract

In the title compound, 2C_4_H_7_N_2_^+^·C_4_H_2_O_4_^2−^·2H_2_O, the asymmetric unit consists of one 2-methyl­imidazolium cation, half a fumarate dianion and one water mol­ecule. There is an inversion center at the mid-point of the central C—C bond of the fumarate anion. In the crystal structure, mol­ecules are linked into a three-dimensional network by inter­molecular N—H⋯O, O—H⋯O and weak C—H⋯O hydrogen bonds. In addition, there are weak π–π stacking inter­actions with centroid–centroid distances of 3.640 (1) Å.

## Related literature

For background information on cocrystals, see: Aakeröy & Salmon (2005 ▶); Aakeröy *et al.* (2007 ▶); Childs & Hardcastle (2007 ▶); Childs *et al.* (2007 ▶).
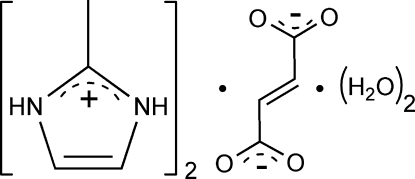


## Experimental

### 

#### Crystal data

2C_4_H_7_N_2_^+^·C_4_H_2_O_4_^2−^·2H_2_O
*M* *_r_* = 316.32
Monoclinic, 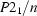

*a* = 8.3912 (8) Å
*b* = 7.3195 (7) Å
*c* = 14.2475 (13) Å
β = 106.624 (2)°
*V* = 838.50 (14) Å^3^
*Z* = 4
Mo *K*α radiation
μ = 0.10 mm^−1^
*T* = 294 K
0.20 × 0.10 × 0.04 mm

#### Data collection

Bruker SMART APEX CCD area-detector diffractometer
Absorption correction: multi-scan (*SADABS*; Bruker, 2001 ▶) *T*_min_ = 0.970, *T*_max_ = 0.996
9171 measured reflections
1920 independent reflections
1261 reflections with *I* > 2σ(*I*)
*R*_int_ = 0.036

#### Refinement

*R*[*F* ^2^ > 2σ(*F* ^2^)] = 0.051
*wR*(*F* ^2^) = 0.152
*S* = 1.06
1920 reflections
114 parameters
H atoms treated by a mixture of independent and constrained refinement
Δρ_max_ = 0.25 e Å^−3^
Δρ_min_ = −0.18 e Å^−3^

### 

Data collection: *SMART* (Bruker, 2001 ▶); cell refinement: *SAINT-Plus* (Bruker, 2001 ▶); data reduction: *SAINT-Plus*; program(s) used to solve structure: *SHELXS97* (Sheldrick, 2008 ▶); program(s) used to refine structure: *SHELXL97* (Sheldrick, 2008 ▶); molecular graphics: *PLATON* (Spek, 2009 ▶); software used to prepare material for publication: *PLATON*.

## Supplementary Material

Crystal structure: contains datablocks global, I. DOI: 10.1107/S160053680903952X/lh2915sup1.cif

Structure factors: contains datablocks I. DOI: 10.1107/S160053680903952X/lh2915Isup2.hkl

Additional supplementary materials:  crystallographic information; 3D view; checkCIF report

## Figures and Tables

**Table 1:** Hydrogen-bond geometry (Å, °)

*D*—H⋯*A*	*D*—H	H⋯*A*	*D*⋯*A*	*D*—H⋯*A*
N2—H2*A*⋯O3^i^	0.90 (2)	1.79 (2)	2.682 (2)	172 (2)
O3—H3*B*⋯O2^ii^	0.80 (4)	1.94 (4)	2.733 (2)	177 (4)
C5—H5⋯O1^iii^	0.93	2.38	3.308 (3)	175
N1—H1*A*⋯O1	0.96 (2)	1.71 (2)	2.668 (2)	173 (2)
O3—H3*A*⋯O2	0.81 (4)	1.94 (4)	2.742 (2)	176 (4)

Symmetry codes: (i) 
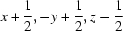
; (ii) 
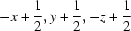
; (iii) 
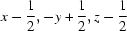
.

**Fig. 1.**
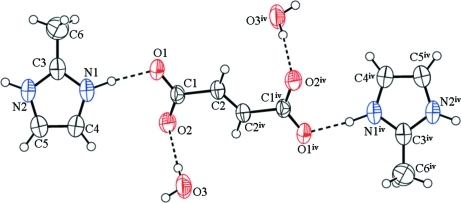
Molecular structure of (I), showing the atom-numbering scheme. Displacement ellipsoids are drawn at the 50% probability level. H-bonds are shown in dashed lines. (Symmetry code (iv): 1-x, -y, 1-z).

**Fig. 2.**
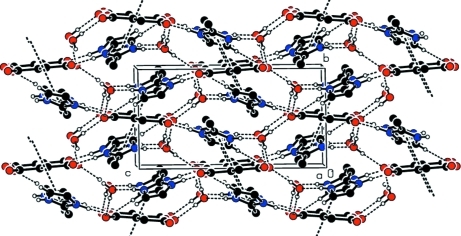
Part of the crystal structure of (I), showing the formation of the three-dimensional network by N-H···O, O-H···O and C-H···O hydrogen-bonds and π–π stacking interactions shown as dashed lines. Hydrogen atoms not involved in the motif have been omitted for clarity.

## supplementary crystallographic information

### Comment

Studies on cocrystals or organic salts have been expanded rapidly in recent years owing to their potential application in active pharmaceutical ingredients (Aakeröy *et al.*, 2007; Childs *et al.*, 2007; Hilds & Hardcastle, 2007). Herein, the crystal structure an organic salt formed by the reaction of 2-methylimidazole and fumaric acid is reported.

In the title compound (I), the asymmetric unit is composed of one 2-methylimidazolium cation, half a fumarate dianion and one water molecule. There is an inversion center at the midpoint of the C2-C2(1-x, -y, 1-z) bond. The title complex can be regarded as an organic salt according to Aakeröy & Salmon (2005). The fumaric acid molecule is deprotonated, with both the protons transferred to the imidazole N atom leading to each a fumarate dianion and an imidazolium cation (Fig.1), which can be evidenced to some extent by the delocaliztion of the carboxyl C-O bonds (C1-O1 1.249 (2)Å, C1-O2 1.253 (2)Å) and the imidazolium C-N bonds (C3-N1 1.325 (2)Å, C3-N2 1.332 (2)Å).

In the crystal packing, by a combination of two N-H···O, two O-H···O and one C-H···O hydrogen bonds (Table 1) and one π-π interaction [Cg···Cg(1-x, 1-y, -z) = 3.640 (1)Å, Cg is the centroid defined by atoms C3-C5/N1/N2], molecules in (I) are linked into a three-dimensional network (Fig.2).

### Experimental

All the reagents and solvents were used as obtained without further purification. A 1:2 molar amounts of fumaric acid (0.2 mmol, 23.2 mg) and 2-methyl-imidazole (0.4 mmol, 32.8 mg) were dissolved in 95% methanol (10 ml). The resulting solution was kept in air for one week. Plate crystals of (I) suitable for single-crystal X-ray diffraction analysis were grown by slow evaporation of the solution at the bottom of the vessel.

### Refinement

H atoms bonded to C atoms were located in difference maps and subsequently treated in a riding-model approximation, with C–H = 0.93 Å (aromatic), 0.96Å (methyl), *U*_iso_(H) = 1.2*U*_eq_( aromatic C) and 1.5*U*_eq_(methyl C). H atoms bonded to N and O atoms were also found in difference maps and their distances were refined freely (see Table 1 for the distances), and the *U*_iso_(H) values being set k times of their carrier atoms ( k = 1.2 for N1 and 1.5 for O atoms and N2)

### Crystal data

**Table:**

2C_4_H_7_N_2_^+^·C_4_H_2_O_4_^2^^−^·2H_2_O	*F*(000) = 336
*M**_r_* = 316.32	*D*_x_ = 1.253 Mg m^−^^3^
Monoclinic, *P*2_1_/*n*	Mo *K*α radiation, λ = 0.71073 Å
Hall symbol: -P 2yn	Cell parameters from 430 reflections
*a* = 8.3912 (8) Å	θ = 3.0–22.0°
*b* = 7.3195 (7) Å	µ = 0.10 mm^−^^1^
*c* = 14.2475 (13) Å	*T* = 294 K
β = 106.624 (2)°	Plate, colorless
*V* = 838.50 (14) Å^3^	0.20 × 0.10 × 0.04 mm
*Z* = 4	

### Data collection

**Table:**

Bruker SMART APEX CCD area-detector diffractometer	1920 independent reflections
Radiation source: fine focus sealed Siemens Mo tube	1261 reflections with *I* > 2σ(*I*)
graphite	*R*_int_ = 0.036
0.3° wide ω exposures scans	θ_max_ = 27.5°, θ_min_ = 2.6°
Absorption correction: multi-scan (*SADABS*; Bruker, 2001)	*h* = −10→10
*T*_min_ = 0.970, *T*_max_ = 0.996	*k* = −9→9
9171 measured reflections	*l* = −18→18

### Refinement

**Table:**

Refinement on *F*^2^	Primary atom site location: structure-invariant direct methods
Least-squares matrix: full	Secondary atom site location: difference Fourier map
*R*[*F*^2^ > 2σ(*F*^2^)] = 0.051	Hydrogen site location: inferred from neighbouring sites
*wR*(*F*^2^) = 0.152	H atoms treated by a mixture of independent and constrained refinement
*S* = 1.06	*w* = 1/[σ^2^(*F*_o_^2^) + (0.071*P*)^2^ + 0.1419*P*] where *P* = (*F*_o_^2^ + 2*F*_c_^2^)/3
1920 reflections	(Δ/σ)_max_ = 0.027
114 parameters	Δρ_max_ = 0.25 e Å^−^^3^
0 restraints	Δρ_min_ = −0.18 e Å^−^^3^

### Special details

**Table:**

Geometry. All esds (except the esd in the dihedral angle between two l.s. planes) are estimated using the full covariance matrix. The cell esds are taken into account individually in the estimation of esds in distances, angles and torsion angles; correlations between esds in cell parameters are only used when they are defined by crystal symmetry. An approximate (isotropic) treatment of cell esds is used for estimating esds involving l.s. planes.
Refinement. Refinement of F^2^ against ALL reflections. The weighted R-factor wR and goodness of fit S are based on F^2^, conventional R-factors R are based on F, with F set to zero for negative F^2^. The threshold expression of F^2^ > 2sigma(F^2^) is used only for calculating R-factors(gt) etc. and is not relevant to the choice of reflections for refinement. R-factors based on F^2^ are statistically about twice as large as those based on F, and R- factors based on ALL data will be even larger.

### Fractional atomic coordinates and isotropic or equivalent isotropic displacement parameters (Å^2^)

**Table:**

	*x*	*y*	*z*	*U*_iso_*/*U*_eq_	
C1	0.5386 (2)	0.0238 (2)	0.37212 (12)	0.0401 (4)	
C2	0.5606 (2)	0.0086 (2)	0.48028 (11)	0.0428 (5)	
H2	0.6685	0.0117	0.5220	0.051*	
C3	0.6978 (3)	0.1228 (2)	0.10113 (13)	0.0471 (5)	
C4	0.4614 (3)	0.2353 (3)	0.11177 (14)	0.0539 (5)	
H4	0.3750	0.2682	0.1372	0.065*	
C5	0.4689 (3)	0.2673 (3)	0.02039 (14)	0.0516 (5)	
H5	0.3895	0.3266	−0.0294	0.062*	
C6	0.8635 (3)	0.0358 (3)	0.12642 (17)	0.0702 (7)	
H6A	0.9432	0.1142	0.1698	0.105*	
H6B	0.8958	0.0159	0.0678	0.105*	
H6C	0.8593	−0.0791	0.1581	0.105*	
N1	0.6039 (2)	0.1456 (2)	0.16080 (11)	0.0517 (5)	
H1A	0.633 (2)	0.101 (3)	0.2268 (16)	0.062*	
N2	0.6165 (2)	0.1953 (2)	0.01497 (11)	0.0482 (5)	
H2A	0.654 (3)	0.191 (3)	−0.0380 (18)	0.074 (7)*	
O1	0.66885 (18)	0.0373 (2)	0.34674 (9)	0.0569 (4)	
O2	0.39407 (17)	0.0221 (2)	0.31492 (8)	0.0556 (4)	
O3	0.2028 (3)	0.3054 (3)	0.34768 (13)	0.1009 (8)	
H3B	0.172 (5)	0.371 (6)	0.301 (3)	0.151*	
H3A	0.256 (5)	0.221 (5)	0.335 (3)	0.151*	

### Atomic displacement parameters (Å^2^)

**Table:**

	*U*^11^	*U*^22^	*U*^33^	*U*^12^	*U*^13^	*U*^23^
C1	0.0536 (12)	0.0402 (9)	0.0280 (8)	0.0051 (8)	0.0144 (8)	0.0001 (7)
C2	0.0490 (12)	0.0515 (11)	0.0276 (9)	0.0012 (9)	0.0102 (7)	0.0001 (7)
C3	0.0631 (13)	0.0426 (10)	0.0375 (9)	−0.0092 (9)	0.0172 (9)	−0.0010 (8)
C4	0.0635 (14)	0.0604 (13)	0.0422 (10)	−0.0028 (11)	0.0224 (10)	0.0006 (9)
C5	0.0624 (14)	0.0513 (11)	0.0402 (10)	−0.0050 (10)	0.0132 (9)	0.0023 (8)
C6	0.0735 (17)	0.0671 (15)	0.0718 (15)	0.0029 (12)	0.0235 (13)	0.0024 (12)
N1	0.0688 (12)	0.0565 (10)	0.0318 (8)	−0.0064 (9)	0.0178 (8)	0.0032 (7)
N2	0.0685 (12)	0.0476 (9)	0.0327 (8)	−0.0111 (8)	0.0210 (8)	−0.0010 (7)
O1	0.0581 (9)	0.0826 (11)	0.0349 (7)	0.0046 (7)	0.0214 (6)	0.0103 (6)
O2	0.0570 (9)	0.0780 (10)	0.0302 (6)	0.0073 (7)	0.0100 (6)	−0.0072 (6)
O3	0.150 (2)	0.1188 (17)	0.0515 (9)	0.0793 (14)	0.0575 (12)	0.0364 (10)

### Geometric parameters (Å, °)

**Table:**

C1—O1	1.249 (2)	C4—H4	0.9300
C1—O2	1.253 (2)	C5—N2	1.368 (3)
C1—C2	1.503 (2)	C5—H5	0.9300
C2—C2^i^	1.301 (3)	C6—H6A	0.9600
C2—H2	0.9300	C6—H6B	0.9600
C3—N1	1.325 (2)	C6—H6C	0.9600
C3—N2	1.332 (2)	N1—H1A	0.96 (2)
C3—C6	1.477 (3)	N2—H2A	0.90 (2)
C4—C5	1.342 (3)	O3—H3B	0.80 (4)
C4—N1	1.368 (3)	O3—H3A	0.81 (4)
			
O1—C1—O2	125.22 (15)	N2—C5—H5	126.8
O1—C1—C2	116.20 (16)	C3—C6—H6A	109.5
O2—C1—C2	118.57 (17)	C3—C6—H6B	109.5
C2^i^—C2—C1	124.6 (2)	H6A—C6—H6B	109.5
C2^i^—C2—H2	117.7	C3—C6—H6C	109.5
C1—C2—H2	117.7	H6A—C6—H6C	109.5
N1—C3—N2	107.42 (19)	H6B—C6—H6C	109.5
N1—C3—C6	126.08 (18)	C3—N1—C4	109.04 (16)
N2—C3—C6	126.5 (2)	C3—N1—H1A	123.6 (13)
C5—C4—N1	107.61 (19)	C4—N1—H1A	127.4 (13)
C5—C4—H4	126.2	C3—N2—C5	109.55 (17)
N1—C4—H4	126.2	C3—N2—H2A	123.4 (15)
C4—C5—N2	106.38 (19)	C5—N2—H2A	127.0 (15)
C4—C5—H5	126.8	H3B—O3—H3A	110 (3)
			
O1—C1—C2—C2^i^	−179.1 (2)	C5—C4—N1—C3	0.1 (2)
O2—C1—C2—C2^i^	0.9 (3)	N1—C3—N2—C5	0.7 (2)
N1—C4—C5—N2	0.3 (2)	C6—C3—N2—C5	−178.31 (19)
N2—C3—N1—C4	−0.5 (2)	C4—C5—N2—C3	−0.6 (2)
C6—C3—N1—C4	178.5 (2)		

Symmetry codes: (i) −*x*+1, −*y*, −*z*+1.

### Hydrogen-bond geometry (Å, °)

**Table:**

*D*—H···*A*	*D*—H	H···*A*	*D*···*A*	*D*—H···*A*
N2—H2A···O3^ii^	0.90 (2)	1.79 (2)	2.682 (2)	172 (2)
O3—H3B···O2^iii^	0.80 (4)	1.94 (4)	2.733 (2)	177 (4)
C5—H5···O1^iv^	0.93	2.38	3.308 (3)	175
N1—H1A···O1	0.96 (2)	1.71 (2)	2.668 (2)	173 (2)
O3—H3A···O2	0.81 (4)	1.94 (4)	2.742 (2)	176 (4)

Symmetry codes: (ii) *x*+1/2, −*y*+1/2, *z*−1/2; (iii) −*x*+1/2, *y*+1/2, −*z*+1/2; (iv) *x*−1/2, −*y*+1/2, *z*−1/2.

## References

[bb1] Aakeröy, C. B., Fasulo, M. E. & Desper, J. (2007). *Mol. Pharm.* **4**, 317–322. 10.1021/mp060126o 17497799

[bb2] Aakeröy, C. B. & Salmon, D. J. (2005). *CrystEngComm*, **7**, 439–448. 10.1039/b811322j PMC2748950 20046916

[bb3] Bruker (2001). *SAINT-Plus*, *SMART* and *SADABS* Bruker AXS, Inc., Madison, Wisconsin, USA.

[bb4] Childs, S. L. & Hardcastle, K. I. (2007). *Cryst. Growth Des.* **7**, 1291–1304.

[bb5] Childs, S. L., Stahly, G. P. & Park, A. (2007). *Mol. Pharm.* **4**, 323–338. 10.1021/mp0601345 17461597

[bb6] Sheldrick, G. M. (2008). *Acta Cryst.* A**64**, 112–122. 10.1107/S0108767307043930 18156677

[bb7] Spek, A. L. (2009). *Acta Cryst.* D**65**, 148–155. 10.1107/S090744490804362X PMC2631630 19171970

